# Development of an Analysis Pipeline Characterizing Multiple Hypervariable Regions of 16S rRNA Using Mock Samples

**DOI:** 10.1371/journal.pone.0148047

**Published:** 2016-02-01

**Authors:** Jennifer J. Barb, Andrew J. Oler, Hyung-Suk Kim, Natalia Chalmers, Gwenyth R. Wallen, Ann Cashion, Peter J. Munson, Nancy J. Ames

**Affiliations:** 1 Mathematical and Statistical Computing Laboratory, Center for Information Technology, National Institutes of Health, Bethesda, Maryland, United States of America; 2 Bioinformatics and Computational Biosciences Branch, Office of Cyber Infrastructure and Computational Biology, National Institute of Allergy and Infectious Diseases, National Institutes of Health, Bethesda, Maryland, United States of America; 3 National Institute of Nursing Research, National Institutes of Health, Bethesda, Maryland, United States of America; 4 National Institute of Dental and Craniofacial Research, National Institutes of Health, Bethesda, Maryland, United States of America; 5 Clinical Center Nursing Department, National Institutes of Health, Bethesda, Maryland, United States of America; International Atomic Energy Agency, AUSTRIA

## Abstract

**Objectives:**

There is much speculation on which hypervariable region provides the highest bacterial specificity in 16S rRNA sequencing. The optimum solution to prevent bias and to obtain a comprehensive view of complex bacterial communities would be to sequence the entire 16S rRNA gene; however, this is not possible with second generation standard library design and short-read next-generation sequencing technology.

**Methods:**

This paper examines a new process using seven hypervariable or V regions of the 16S rRNA (six amplicons: V2, V3, V4, V6-7, V8, and V9) processed simultaneously on the Ion Torrent Personal Genome Machine (Life Technologies, Grand Island, NY). Four mock samples were amplified using the 16S Ion Metagenomics Kit^™^ (Life Technologies) and their sequencing data is subjected to a novel analytical pipeline.

**Results:**

Results are presented at family and genus level. The Kullback-Leibler divergence (D_KL_), a measure of the departure of the computed from the nominal bacterial distribution in the mock samples, was used to infer which region performed best at the family and genus levels. Three different hypervariable regions, V2, V4, and V6-7, produced the lowest divergence compared to the known mock sample. The V9 region gave the highest (worst) average D_KL_ while the V4 gave the lowest (best) average D_KL_. In addition to having a high D_KL_, the V9 region in both the forward and reverse directions performed the worst finding only 17% and 53% of the known family level and 12% and 47% of the genus level bacteria, while results from the forward and reverse V4 region identified all 17 family level bacteria.

**Conclusions:**

The results of our analysis have shown that our sequencing methods using 6 hypervariable regions of the 16S rRNA and subsequent analysis is valid. This method also allowed for the assessment of how well each of the variable regions might perform simultaneously. Our findings will provide the basis for future work intended to assess microbial abundance at different time points throughout a clinical protocol.

## Background

**Fig 1:**
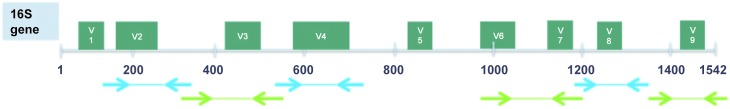
Schematic of 16S rRNA gene and primer targets. Schematic of 16s gene and location of two primer sets from the Ion 16S^™^ Metagenomics Kit *. This kit is composed of two sets of primers in separate tubes targeting seven hypervariable regions along the 16s gene. Primer sets in tube one, represented by blue arrows, shows locations of primers for V2, V4, and V8. Primer sets in tube two, represented by green arrows, shows locations of primer for V3, V6-7 and V9. Sequencing using the Ion Torrent machine is bidirectional, not paired primers. One primer targets two regions, V6 and V7. (* Image is owned by Life Technologies Corporation, www.lifetechnologies.com, copied from https://www.lifetechnologies.com/content/dam/LifeTech/Documents/PDFs/Ion-16S-Metagenomics-Kit-Software-Application-Note.pdf © 2015 Thermo Fisher Scientific, Inc. Used with permission.)

Using the 16S rRNA gene and the advances in high-throughput sequencing techniques or next-generation sequencing (NGS), it is now possible to identify bacteria by their genetic signature [1, 2]. The 16S rRNA gene is composed of approximately 1542 base pairs (Fig 1) and contains nine hypervariable regions surrounded by conserved regions [3]. With the advent of NGS, researchers are able to assign taxonomy by designing primers that targets a particular hypervariable region or multiple consecutive regions. Initially, the entire 16S gene was sequenced using the chain-termination method or Sanger sequencing [4]. This method allowed the sequencing of long DNA strands, often longer than 500 base pairs (bp). However, the Sanger method is expensive and required days to complete. With the advent of short-read NGS, only part of the 16S gene can be sequenced, up to 400 base pairs [5]. Newer third generation sequencing technology can sequence strands longer than 1400 bp, but this technology is still relatively novel or expensive that make it less than ideal for 16S sequencing based taxonomic assignment at this time [6,7].

Advanced NGS has not only revolutionized research and made it possible to test many hypotheses that previously had been impossible but also established a new “gold standard” for bacterial identification [8, 9]. However, an interesting and complex question related to the 16S rRNA gene is: Which hypervariable (V) region(s) of the gene supply sufficient sequence diversity to identify the most bacteria accurately [10, 11]. Inherent biases arise depending on the region selected. For example, it is known that specific hypervariable regions are more likely to identify certain bacteria [12–15]. Such biases might distort the comprehensive view of complex bacterial communities.

The oral microbiome, because of its diversity and location at the entrance into the aero digestive tract, is a complex community and the selection of which hypervariable region(s) of the 16S gene to use, is a crucial step. Many attempts at identifying the most robust hypervariable region have been published [14,16–21]. In the oral microbiome, researchers have used hypervariable regions V1-2 or V3 in studying saliva [22–23]. In examining 18 different sites in the human microbiome including many in the oral microbiome, Huse et al. examined V1-3 and V3-5 and found comparable results when using these two sequencing sets [24]. An earlier study by Lazarevic et al. used V5 to identify oral bacteria from multiple oral sites [25]. Kumar and colleagues collected subgingival plaque specimens from smokers with periodontal disease and compared three different regions of the 16S rRNA gene (V1-3, V4-6 and V7-9) and Sanger sequencing [19]. These researchers concluded that it was important to use V1-3 and V7-9 to capture results which are similar to Sanger sequencing. Perhaps then, there is not just one region but rather several regions that should be selected based on the type of bacteria one is hoping to identify. While researchers tend to favor the V4 hypervariable region, there is a lack of consensus regarding which V region to use and no consistent hypervariable region is employed in all microbiome studies [16, 26–29].

In this paper we present the results of a novel approach simultaneously sequencing 6 of the hypervariable regions of the 16S rRNA gene in mock samples. The Ion 16S Metagenomic Kit ^™^ (Catalog no. A26216 Life Technologies, Grand Island, NY) was recently offered by the company to be used with the Ion Torrent Personal Genome Machine (Life Technologies, Grand Island, NY). The kit includes 6 proprietary primers designed to target V2, V4 and V8 in one multiplex PCR reaction and in a second reaction, to target V3, V6-7, and V9 [30]. To date, the only analytical pipeline suggested for use with this kit, is the Ion Reporter software by the Life Technologies Company. The goal of this work is to develop an alternative method utilizing open access tools such as QIIME, Mothur and UPARSE for downstream processing and hypothesis testing.

## Methods

### Collection of microbial mock communities

Mock samples Human Microbiome Project: Genomic DNA from Microbial Mock Community B (Staggered, Low Concentration), v5.2L, for 16S rRNA Gene Sequencing, HM-783D, Genomic DNA from Microbial Mock Community B (Staggered, High Concentration), v5.2H, for Whole Genome Shotgun Sequencing, HM-277D, Genomic DNA from Microbial Mock Community B (Even, High Concentration), v5.1H, for Whole Genome Shotgun Sequencing, HM-276D, Genomic DNA from Microbial Mock Community B (Even, Low Concentration), v5.1L, for 16S RNA Gene Sequencing, HM-782D. The mock communities each contained 20 different common bacterial strains [31]. Gram positive and negative bacteria were included. The Even samples (Even High and Even Low) contained equal numbers of ribosomal RNA operon counts across all bacteria with Even High containing 10^6^ operons per bacteria and Even Low with 10^5^. The other two samples (Staggered High and Staggered Low) contained staggered numbers of ribosomal RNA operon counts differing by bacteria with the Staggered High sample ranging from 10^3^ to 10 ^7^ operons and the staggered low sample ranging from 10^3^ to 10^6^ operons. The four samples are accessible through NCBI's Sequence Read Archive (SRA), under study accession number SUB1054354.

### 16S Metagenomics Kit Sequencing using Ion Torrent PGM

The 16S region was amplified with 16S Ion Metagenomics Kit ^™^ (Life Technologies) by 2 separate PCR reactions using primer set V2, V4, V8 and V3, V6-7, V9. Equal volumes of V2, V4, V8 and V3, V6-7, V9 amplification reactions were combined. Fifty nanograms of combined amplicons were processed to make the DNA library using Ion Plus Fragment Library Kit ^™^ and Ion Xpress Barcodes Adapters, 1–16 ^™^ (Life Technologies, Grand Island, NY). Adapter-ligated and nick-repaired DNA was amplified with the following steps: 1 cycle of 95°C for 5 min; 5 cycles of 95°C for 15sec, 58°C for 15 sec, 70°C for 1 min; hold at 4°C. Each step was followed by purification using 1.4 volumes of Agencourt AMPure beads (Beckman Coulter, Inc, Atlanta, Georgia) and eluted in low Tris-EDTA buffer. Size and quantity of processed libraries were evaluated with DNA high sensitivity kit in 2100 Bioanalyzer (Agilent Technologies, Santa Clara, CA.). Each sample was adjusted to 26 picomolar concentration. Equal volumes of all 4 samples were combined and processed with One-Touch 2 and One-Touch ES systems (Life Technologies, Grand Island, NY) according to the manufacturer’ instructions. Sequencing was performed on the Ion Personal Genome Machine (PGM) using 400-bp kit and 316 v2 chip. Base calling and run demultiplexing were performed by Torrent Suite version 4.4.2 (Life Technologies, Grand Island, NY) with default parameters. FileExporter version 4.4.0. 0 (Life Technologies, Grand Island, NY) was used to generate demultiplexed fastq files for each sample. Mean read length for both forward and reverse reads ranged between 235bp to 238bp for all four samples.

### Data processing and preparation

**Fig 2:**
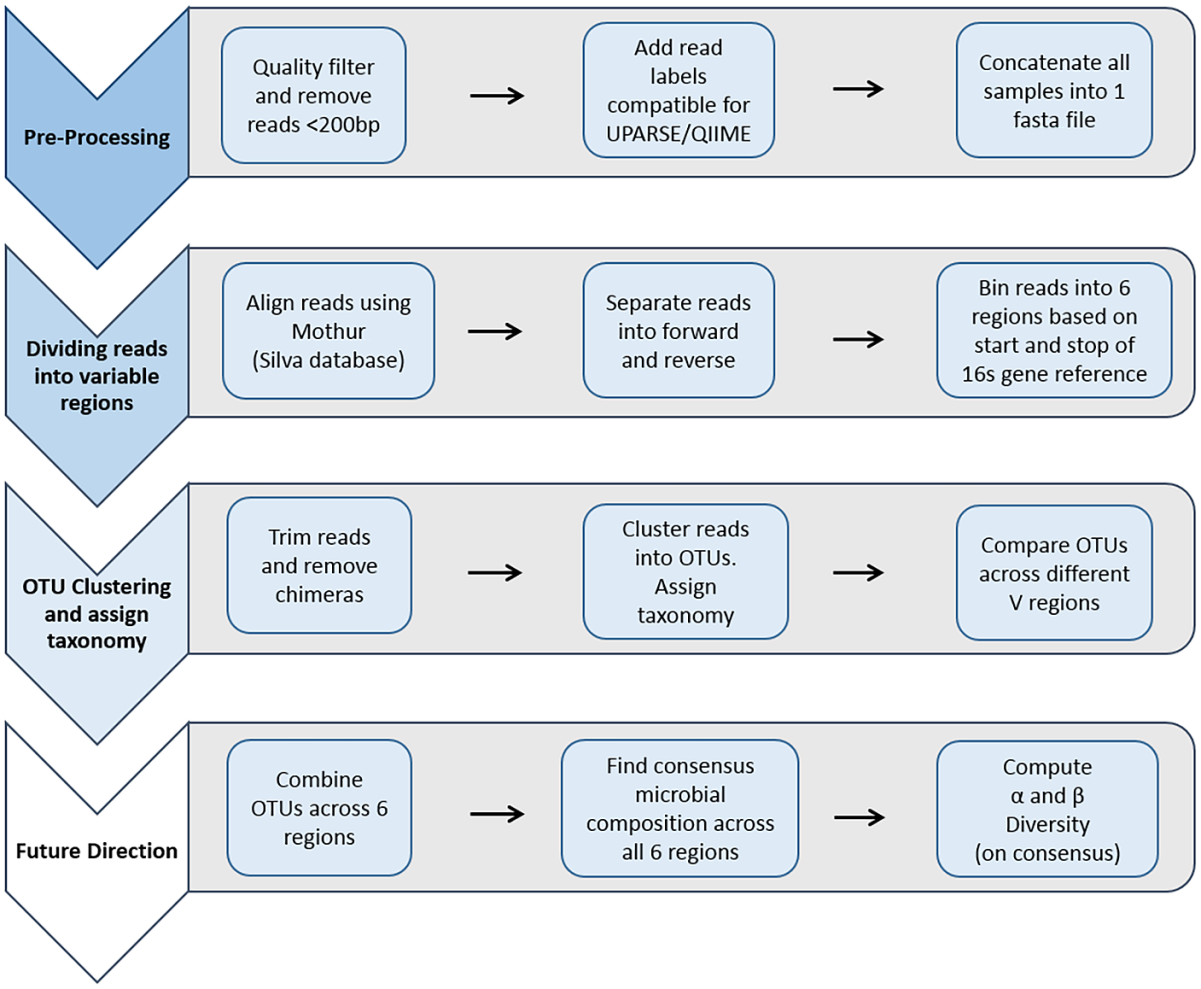
Data Processing Pipeline. Workflow of data processing pipeline using Ion 16S Metagenomics Kit. Step 1, Pre-processing of the data consists of quality filtering, read ID editing and concatenating reads into one file. Step 2, creating subsets of reads into appropriate targeted variable regions consists first of using Mothur to align the reads, then separating reads into forward and reverse based on their alignment into 16S gene coordinates. This last step allows reads to be binned into their appropriate targeted region. Step 3, uses a mixture of QIIME and UPARSE for OTU clustering and taxonomy assignment. Step 4, discusses future directions in order to come up with a consensus table taking into account bacteria found from each variable region and computing α and β diversity.

The data pipeline can be described in 3 steps for this work (Fig 2). Pre-processing includes quality filtering and length filtering, adding read labels in order to mimic non-demultiplexed data for downstream analysis, and concatenating reads into one file. The second step involves dividing reads into 6 subsets of the 6 hypervariable regions. This step begins with aligning the reads to the Silva Database using Mothur, separating reads into forward and reverse, and binning reads based on start and stop coordinate from the Mothur alignment [32–33]. The third step, Operational Taxonomic Units (OTU) clustering and taxonomic assignment, includes trimming reads and removing chimeras, clustering reads into OTUs and assigning taxonomy using Quantitative Insights into Microbial Ecology (QIIME) [34]. Finally, OTUs were compared across different V regions. Future work will be to develop a consensus OTU table, if possible, taking into account OTUs from each region.

### Pre-processing

Reads were quality filtered using a fastq filter script from the USEARCH suite of tools (fastq_filter) and removed if they were shorter than 200bp and had more than 1 total expected number of errors for all bases in the read (Fig 2) [35]. The total expected number of errors is the sum of the error probabilities (more information can be found in the USEARCH manual). Read IDs were edited and formatted to match the downstream processing pipeline using UPARSE and QIIME so that a read ID contained “Ex##;barcodelabel = [sample name];” where ‘##’ is a number in numerical order and ‘sample name’ is the sample identifier [34,36,37]. Once reads from each sample were quality filtered and IDs edited, the data were concatenated so that one fasta file contains reads from all samples in the study to mimic non-demultiplexed data.

### Separating reads into different variable regions

**Fig 3:**
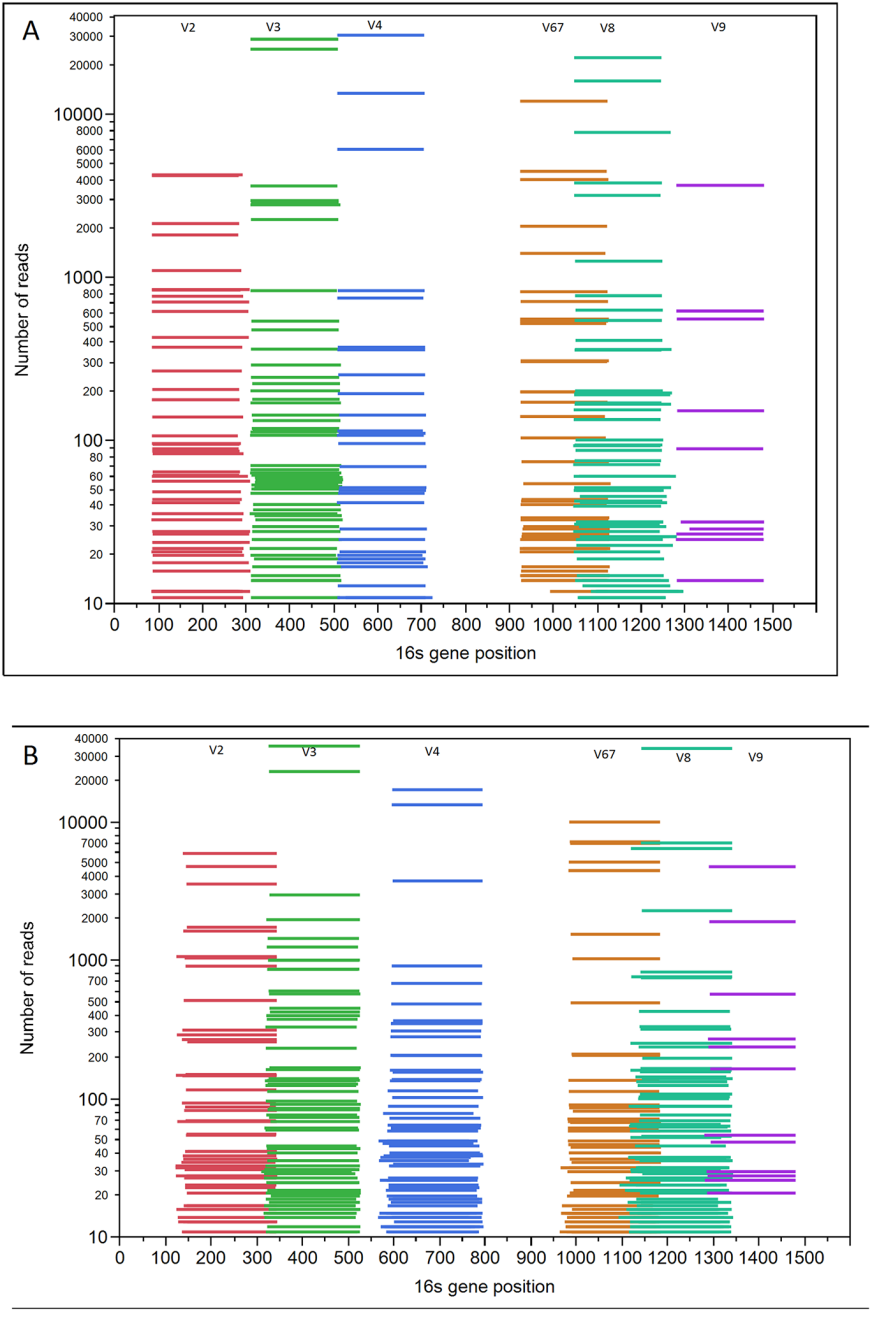
Aligned Reads by Region. Coverage of aligned forward (A) and reverse (B) reads. X-axis shows the position along the 16S rRNA gene using *Streptococcus mutans* (GenBank accession DQ677761) as the reference and the y-axis shows the number of reads giving the same start and stop position. Colors of the lines indicate which variable region the read was assigned to given the aligned coordinates. Colors correspond to reads assigned to 1 of 6 subsets of reads for OTU picking; V2 (red), V3 (green), V4 (blue), V6-7 (orange) and V8 (blue-green) and V9 (purple).

Because reads from libraries generated from the 16S Metagenomics Kit contain 6 primer pairs targeting 7 different variable regions (one primer pair targets V6 and V7 thus this paper will refer to 6 regions), the first step was to separate the reads into their respective targeted regions before OTU picking. In order to do this, we aligned the full set of reads with the Mothur script align.seqs using Silva as the reference database (www.mothur.org/, Silva version 119, database downloaded August 2014). All default parameters were used along with the option ‘flip = true’ [32]. The addition of this parameter will evoke the script to print out the read ids that aligned better after reverse complementing. The aligned fasta file was then submitted to an in-house Perl script that counts the number of reads with the same start and stop alignment position along the reference database. The reference database coordinates were translated to the 16S rRNA gene coordinates using one aligned reference sequence, *Streptococcus mutans* (Genbank Accession: DQ677761). The forward and reverse reads were then visualized along the translated 16S rRNA gene coordinates (Fig 3) and reads were grouped into their corresponding variable regions based on where they aligned (Table 1). The script, filterbyname.sh from BBMap tools was used to make 12 different subsets (forward and reverse for each of 6 regions) of reads by read IDs [38].

**Table 1:** Division of Reads.

	Direction	Start	Stop	Number of reads	Number of OTUs
V2	forward		< = 350	20,835	23
V2	reverse		< = 370	24,649	23
V3	forward	> = 200	< = 600	75,304	19
V3	reverse	> = 250	< = 550	77,267	19
V4	forward	> = 450	< = 800	55,038	21
V4	reverse	> = 500	< = 850	41,404	20
V6-7	forward	> = 850	< = 1180	29,995	22
V6-7	reverse	> = 900	< = 1200	40,023	24
V8	forward	> = 1000	< = 1370	61,559	18
V8	reverse	> = 1050	< = 1360	58,252	20
V9	forward	> = 1200		5,354	3
V9	reverse	> = 1250		8,212	10

Table showing division of reads into respective variable regions. Binning start and stop coordinates for both forward and reverse reads into their respective regions. Total number of rewards per region shown in column 5 “Number of reads”. Total number of OTUs after OTU picking for each region shown in column 6 “Number of OTUs”.

Forward reads (Fig 3A) were grouped into variable regions by the following: V2 stop coordinates had to be < = 350, V3 reads had to align between 200–600, V4 reads had to align between 450 bp-800 bp, V6-7 reads had to align between 850–1180, V8 reads had to align between 1000–1370 and V9 start coordinates had to start at least by 1200. Reverse reads (Fig 3B) were grouped into variable regions by the following: V2 stop coordinates had to be < = 370, V3 reads had to align between 250–550, V4 reads had to align between 500 bp-850 bp, V6-7 reads had to align between 900–1200, V8 reads had to align between 1050–1360 and V9 start coordinates had to start at least by 1250.

### Classification and taxonomic assignment per region

The data analysis workflow was adapted from a pipeline published by the Brazilian Microbiome Project specific to Ion Torrent data, which incorporates tools from QIIME and UPARSE [39,40]. The pipeline was run 12 separate times in order to create OTU tables for each of the 6 regions from both forward and reverse reads. Because the primer sequences were not published by the company, the primer trimming step involved globally removing the first 20 base pairs, leaving median read lengths of about 200–220 bp in length. Reads were dereplicated, abundance sorted and singleton sequences were discarded using the UPARSE pipeline. OTU clustering was performed using UPARSE (cluster_otus). Chimera checking due to PCR artifacts was performed using the UCHIME algorithm using the “Gold” database from ChimeraSlayer as the reference [41,42]. Reads were mapped back to the OTU database using USEARCH7 with a minimum identity of at least 97%. OTUs were labeled using a UPARSE Python script (uc2otutab.py). Taxonomy assignment was performed using QIIME (assign_taxonomy.py).

**Fig 4:**
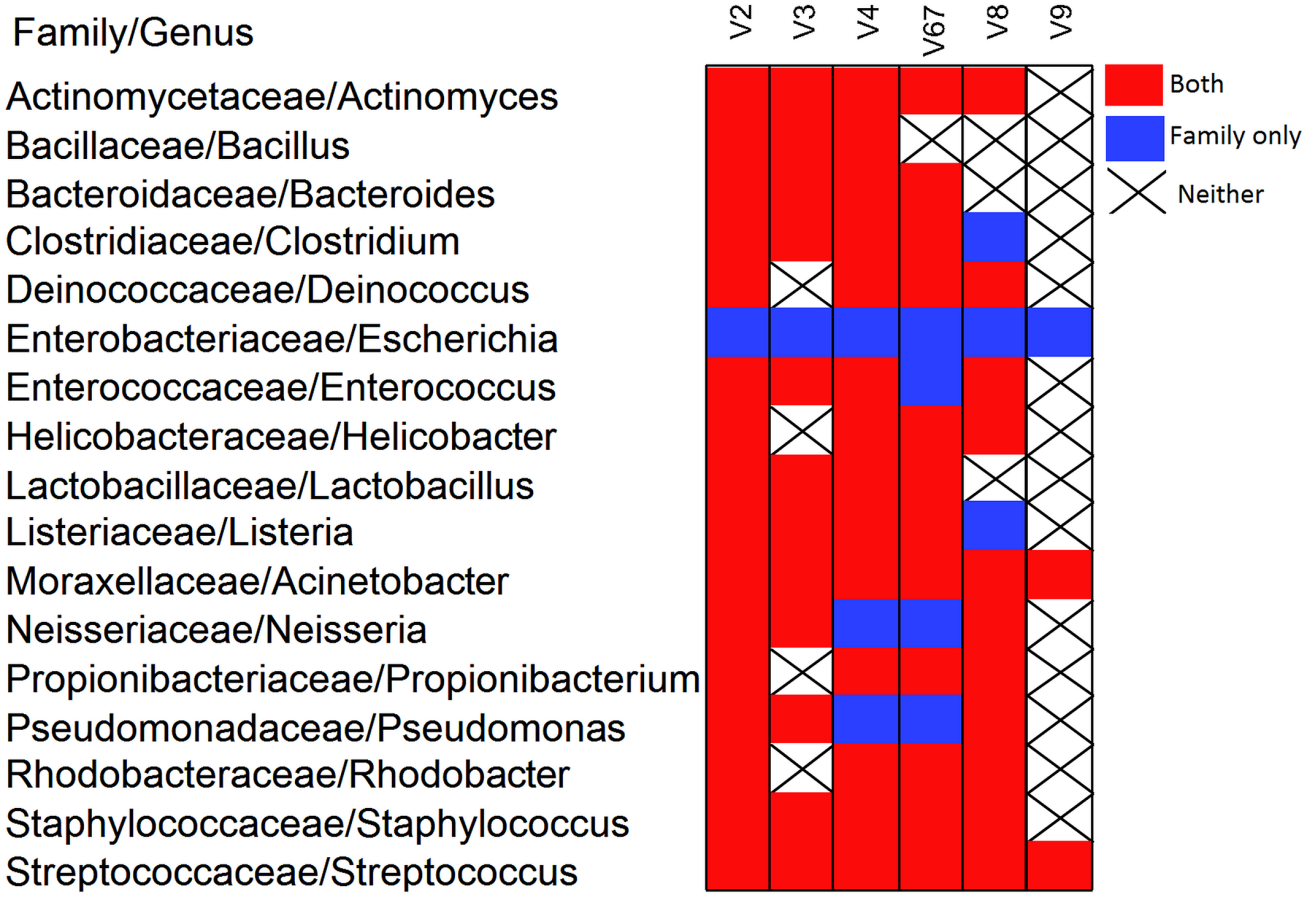
Incidence map showing bacteria found at family and genus level. Incidence map showing whether bacteria was found in the forward reads at both the family and genus (red), at the family only (blue) or not found at family or genus level (black X) for each of the 6 regions.

In order to compare reads assigned to each region against the known mock community, for family level analysis, OTU counts were determined by summing over multiple OTUs assigned to the same family and for genus level analysis the same was performed by summing over multiple OTUs assigned to the same genus level. Any OTU not classified up to family level and/or genus was labeled “Unassigned”. The presence of bacteria in any of the four mock samples for each region and for both forward and reverse was recorded and reported at the family and genus level (Fig 4). Reverse reads are shown in S1 Fig.

### Comparison of multiple variable regions

**Fig 5:**
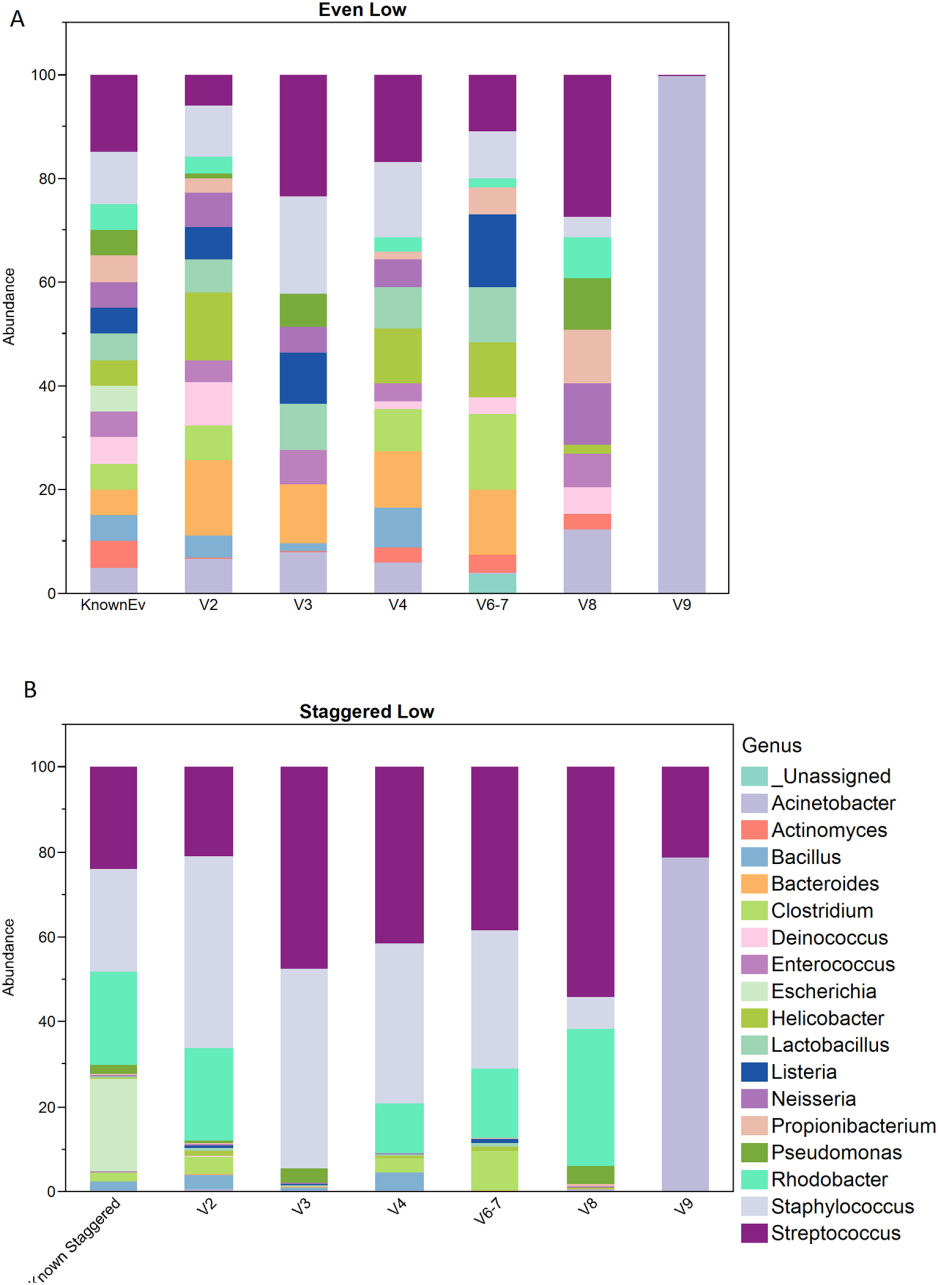
Family Level Bacterial Abundance. Bacterial abundance for known mock samples and 6 other variable regions of forward reads. (A) Genus Level Known Even vs. Even Low mock samples, (B) Genus Level Known Staggered vs. Staggered Low mock samples. (Figs A and B in S2 Fig show Even High and Staggered High samples for genus level)

Proportional abundance within each mock sample for each region was determined for both family and genus levels and these proportions were compared to the expected proportions for both the Even and Staggered samples provided by BEI Resources (Manassas, Virginia) [31]. Stacked bar charts at the genus level are shown to illustrate how well the observed proportions for four mock samples, Even High, Even Low, Staggered High and Staggered Low match to the expected Even and expected Staggered. Fig 5 shows Even Low (Fig 5A) and Staggered Low (Fig 5B) for genus level mock samples for each of the 6 regions forward reads. (S2 Fig shows Even High (Fig A in S2 Fig) and Staggered High (Fig B in S2 Fig) forward reads at the genus level). Genus level proportions were determined by first summing up OTU counts that classified up to and beyond genus level and then calculating the sum divided by the total over all genus level counts.

**Fig 6:**
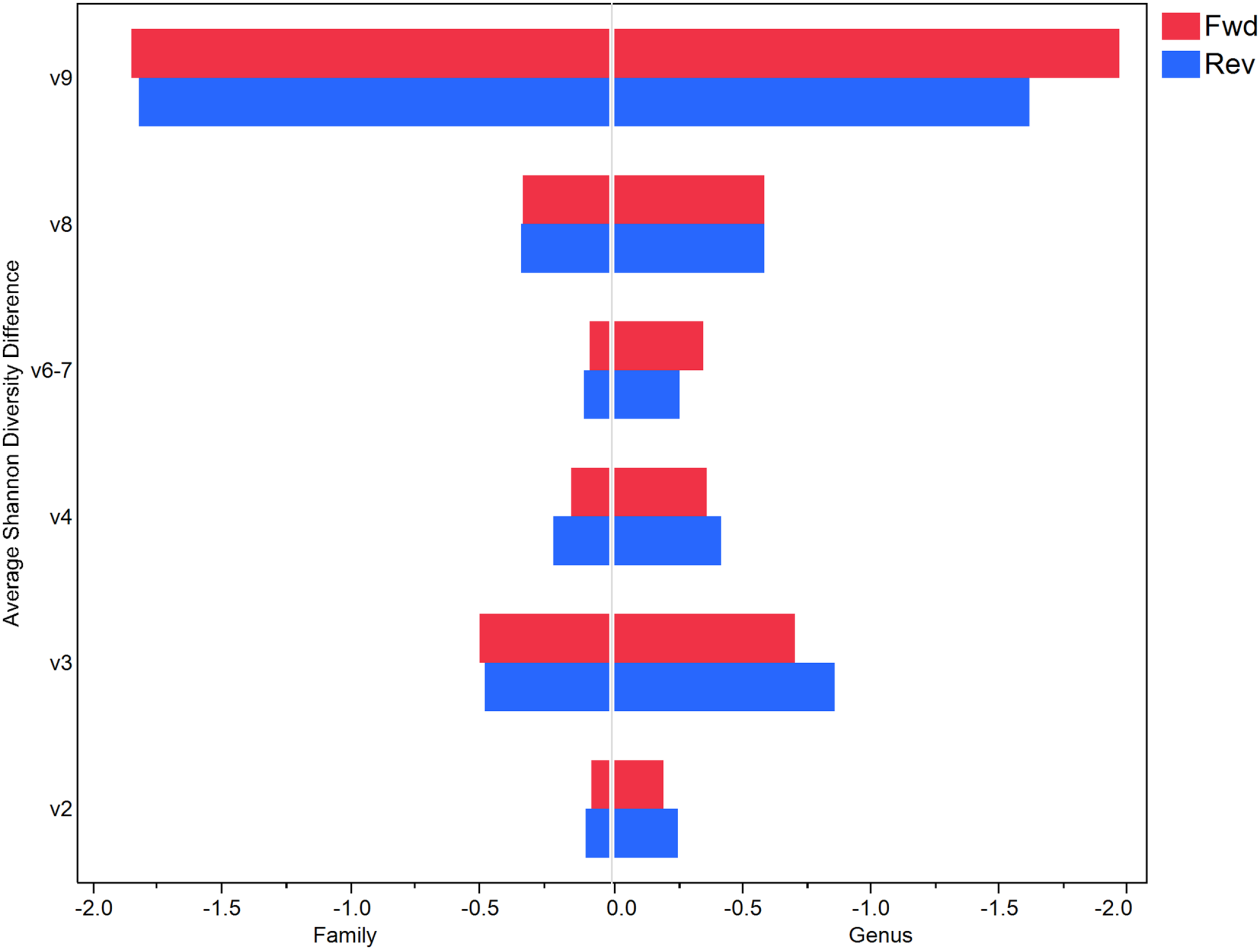
Average Shannon Diversity Difference from known at the Family and Genus level. Average difference between the known Shannon Diversity index vs that calculated for each region at both the family and genus level. Y-axis shows the hypervariable region. X-axis shows the Family (left) and Genus (right) average difference values. Red bars show the average difference in the forward read analysis and the blue bars show the average difference in the reverse read analysis.

In an attempt to see how well the diversity and evenness of the communities for each region for each mock sample matched that of the known, the Shannon Diversity (SD) index was calculated for both the known and observed mock samples at the family and genus level (Table 2) and the difference between the known SD and the average SD across the samples for each region was calculated for both family and genus level (Fig 6). The Shannon Diversity index gives a measure of the species diversity in a given community while taking into account both the abundance and the evenness of species present in a community [43].

**Table 2:** Shannon Diversity Index.

Observed	Known	V2	V2	V3	V3	V4	V4	V6-7	V6-7	V8	V8	V9	V9
		Fwd	Rev	Fwd	Rev	Fwd	Rev	Fwd	Rev	Fwd	Rev	Fwd	Rev
EvHi	2.76	2.68	2.58	2.23	2.31	2.57	2.48	2.52	2.51	2.35	2.32	0.7	0.7
		2.61	2.42	2.11	2.11	2.41	2.28	2.34	2.38	2.13	2.14	0.02	0.13
EvLo	2.76	2.65	2.59	2.24	2.32	2.6	2.49	2.54	2.54	2.37	2.35	0.7	0.7
		2.58	2.43	2.1 2	2.12	2.43	2.29	2.34	2.40	2.16	2.18	0.03	0.13
StHi	1.7	1.64	1.66	1.19	1.18	1.56	1.52	1.78	1.76	1.44	1.43	0.06	0.06
		1.45	1.48	0.90	0.62	1.32	1.33	1.43	1.58	1.15	1.13	0.45	0.89
StLo	1.7	1.67	1.71	1.23	1.16	1.59	1.55	1.77	1.69	1.42	1.41	0.05	0.05
		1.50	1.60	0.94	0.61	1.32	1.34	1.42	1.54	1.12	1.11	0.52	1.28

Shannon diversity index at the family (upper cell) and genus level (lower cell) calculated for four mock samples on known and observed in 6 regions for both forward and reverse.

**Fig 7:**
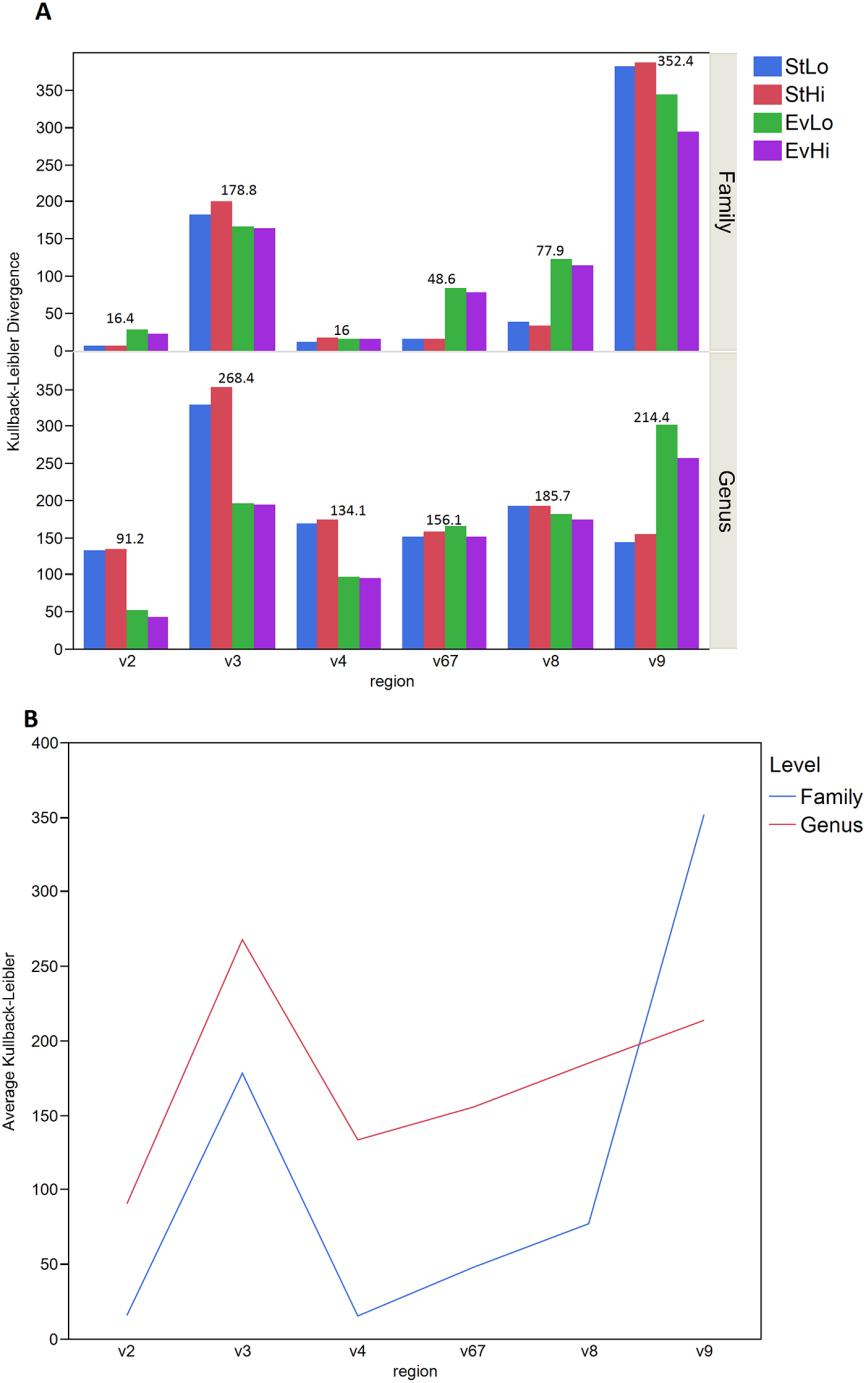
Kullback-Leibler Divergence. (A) Bar graph showing family (upper) and genus (lower) level Kullback-Leibler divergence (y-axis) for 6 regions (x-axis). Known mock compared to observed mock (EvHi, purple; EvLo green; StHi red; StLo blue) for forward reads. The average D_KL_ over all mock samples for each region is shown above the bar charts for a particular region. (B) Average Dkl over 4 mock samples at each region for both family (blue) and genus (red).

The Kullback-Leibler divergence D_KL_ measures the difference between two probability distributions, P and Q. A larger D_KL_ shows a bigger divergence between the two.[44] The Kullback-Leibler divergence was calculated at family and genus level for each observed mock sample versus its respective known mock for each of the 6 regions for both forward (Fig 7A) and reverse reads (S3 Fig). In order to avoid dividing by 0, a constant of 1 was added to the entire set of count data before the proportions were calculated. This addition of the constant of 1 accounted for those cases where a region was unable to find a particular taxonomic group and had a count of 0 or missing entry. Average D_KL_ over all 4 mock samples for each region was calculated for both family and genus and plotted for each region (Fig 7B).

### Comparison of forward and reverse reads

In order to investigate how well forward and reverse reads for each region compared, forward and reverse reads were joined by family level bacteria for each region and total OTU counts were compared in bivariate plots. Bivariate plots for four mock samples and 3 regions, V2, V4 and V6-7 can be seen in S4 Fig.

## Results

### Filtering and separating reads into variable regions

Reads were visualized along the 16S gene of *Streptococcus mutans* (DQ677761) in order to identify boundaries for variable region groupings (Table 1 and Fig 3). As shown in Table 1, V3 was assigned the most reads averaging 76,285 over forward and reverse. Reads targeting V9 had the smallest number of reads averaging at 6,783 over forward and reverse. Start and stop coordinates for region assignments were chosen based on their distribution in Fig 3. Reverse reads are shifted by about 50bp. The total number of reads and OTUs for each region is noted in Table 1, column 5 and 6 respectively.

### Read classification for each mock sample over 6 regions

Classification up to the genus level was investigated for each region in both forward and reverse. Surprisingly, bacteria in the genus *Escherichia* were not identified for all regions in both forward and reverse reads, however the family level Enterobacteriaceae was in fact identified for all regions. Since the mock community composition is known and includes *E*. *coli*, the decision to present both family and genus level data was made for this project. Fig 4 shows the 17 family and genus level bacteria present respectively in the mock samples and whether or not that bacteria was found at both family and genus, family only or not found up to either level for a particular region in any of the four mock samples. Regions giving better resolution than others were V2, V4 and V6-7 while V3, V8 and V9 gave less resolution up to both family and genus level. V4 gave 100% resolution in both forward and reverse for family but gave only 82% resolution for genus, i.e. all 17 bacteria were found at the family level among any of the 4 mock samples and 14/17 bacteria were found at the genus level. V2 gave 100% and 94% resolution in the forward reads for family and genus and found 88% and 76% of the bacteria at the genus in the reverse reads. The V9 region performed poorly at both family and genus level finding less than 60% of the bacteria in any direction. Not noted in the figure is one particular bacteria found that is not present in the mock, *Planococcaceae* for the V8 forward and V3 reverse regions. Forward reads targeted to the V6-7 region had a small percentage of unassigned bacteria. These reads clustered to an OTU that classified only up to order Bacillales.

While we report on family and genus level findings in this study, it is important to note which regions classified the most species correctly. In the forward reads, V2 was able to classify 9 OTUs up to species, V3 was able to classify 2 OTUs up to species, V4 was able to classify 4 OTUs up to species, V6-7 was able to classify 4 OTUs up to species, V8 was able to classify 4 OTUs up to species and V9 did not classify any of its 3 OTUs up to species level. In the reverse reads, V2 was able to classify 6 OTUs up to species, V3 was able to classify 3 OTUs up to species, V4 was able to classify 3 OTUs up to species, V6-7 was able to classify 5 OTUs up to species, V8 was able to classify 5 OTUs up to species and V9 was able to classify 2 OTUs up to species however one of those was misclassified from *Acinetobacter baumannii* to *Acinetobacter rhizosphaera*.

### Differences between forward and reverse

We looked at how different forward and reverse reads are for each region. We first looked at whether a forward or reverse set per region identified each expected bacterial family. We found that for all regions except V6-7 and V9, forward reads found overall more expected bacterial strains than reverse reads (Fig 4 and S1 Fig). In V6-7, V8 and V9, Bacillacea/Bacillus was not found in forward but was found in reverse reads and there were quite a few bacteria that were not found in V9 forward. Interesting to note is that the total read count for the V9 forward set was about 35% less than that for the reverse set which could attribute to this finding. In addition, we looked at the Shannon Diversity for both forward and reverse and compared it to that of the known and found that the SD differences for both forward and reverse within a region were similar to each other (Fig 6 and Table 2). We compared the OTU counts for forward and reverse at the family level for V2, V4 and V6-7 (S4 Fig). The V2 Even samples are less similar than the Staggered samples and namely we see bacteria having higher OTU counts in the reverse than in the forward for V2 (Fig A in S4 Fig). We do see that there were slightly higher total read counts for V2 reverse than for V2 forward. The V4 Even samples are similar (Fig B in S4 Fig), falling along the line of identity but forward tends to have higher OTU counts than reverse. The Staggered V4 samples look very similar. All samples for region V6-7 also look similar with the Even samples having slightly higher OTU counts in the reverse reads. Overall, V4 forward and reverse have the highest agreement when reviewing total number of bacteria identified and in the OTU counts. The V6-7 Even High and Staggered High samples showed slightly higher OTU counts for reverse compared to forward (Fig C in S4 Fig). The Even Low and Staggered Low OTU counts looked similar with many of the counts falling along the line of identity. After reviewing this information, we find that we cannot say definitively that either forward or reverse is better than the other and therefore we will proceed with analyzing reads separately in future work.

### Detectability of bacteria with low operons

The sequenced mock samples provide a theoretical mock community, where DNA from individual species is pooled based on the number of ribosomal RNA (rRNA) operon counts in the respective genomes. In an effort to investigate the detectability threshold (i.e. those bacteria having operon counts at 1,000) of the current sequencing and analytical pipeline, we sequenced a mock sample (Staggered Low) containing bacteria with operons ranging from 1,000 to 1,000,000. There were 5 different species present at the lowest operon level of 1,000, those being *Actinomyces odontolyticus*, *Bacteroides vulgatus*, *Deinococcus radiodurans*, *Enterococcus faecalis* and *Streptococcus pneumonia*. We will discuss four of these five bacteria at the genus level since *Streptococcus* has three species with low to high operon counts. *Actinomyces* was detected only in V4, V6-7 and V8 forward in counts of 3, 2 and 1. The other regions were not able to detect *Actinomyces* in forward. In reverse, the only region able to detect *Actinomyces* was V6-7 with a count of 1. This could be considered not detected depending on optional count filtering. *Bacteroides* was detected in all forward regions except V8 and V9 at counts between 4–9. For reverse regions, *Bacteroides* was detected only in V3, V4 and V6-7 in counts of 8, 4 and 5. *Deinococcus* was detected in all forward regions except V3 and V9 at counts between 2–5. For reverse regions, *Deinococcus* was only detected at counts of 1 for V2 and V4 only. Finally *Enterococcus* was detected in forward regions V3, V4, V6-7 and V8 at counts of 2, 6, 1 and 2. For reverse regions, *Enterococcus* was detected in all regions except V9 at counts ranging from 1–6. These results show that some regions are better at detecting low-level bacteria than others. If a method for generating a consensus table over all 6 regions is obtained, then it is sufficient to say that this method is able to detect even lowly abundant bacteria.

### Comparison of known versus observed mock samples and statistical analysis

Stacked bar charts for the known and observed Even Low and Staggered Low samples shown in Fig 5 gives an illustration of how well a region performed to what was expected. As shown in Fig 5A, regions V2, V4 and V6-7 look most similar to the known even bar chart. Regions V3 and V8 also look similar but appear to be missing some of the bacteria. Region V9 clearly shows the most dissimilarity from what is expected. Interestingly, Fig 5B, Staggered Low, seems to show that regions V2, V4, V6-7 and V8 show the closest similarity to the known while regions V3 and V9 show more dissimilarity. In order to quantitate how well the observed distribution compared to that of the known, the Kullback-Leibler divergence for both the family and genus level data was calculated for the forward and reverse reads and the results are shown in Fig 7A and 7B and S3 Fig. Regions V2, V4, V6-7 and V8 give the lowest D_KL_ for forward in both the family and genus level results while regions V3 and V9 give the highest average D_KL_ over all four mock samples. Fig 7B shows the average D_KL_ for each region and a similar trend across the regions is seen between the family and genus level data. Considering that regions V2 and V4 showed the lowest average D_KL_ over all four samples and found all 17 bacteria at least at the family level, it is worth mentioning that these regions might be the best to use to draw conclusions from for future studies.

In addition to reporting the family level Kullback-Leibler divergence for each sample, we also computed the family level and genus level Shannon Diversity (SD) index in the known mock samples and that for each of the observed mock samples for each region (Table 2) for both forward and reverse reads. As shown in the table, the SD for the known Even samples is 2.76 and that for the Staggered samples is 1.7. In order to visualize how different a SD for a region is from the known SD, we plotted the average difference between the known and mock samples for each region. Fig 6 shows the histogram of these differences for both forward and reverse reads. Interestingly, the forward and reverse bars are very similar suggesting that the diversity is not changed between either the forward and reverse analyses. In addition, the smallest difference is seen in V2, V4 and V6-7 for both the Family and Genus results. The largest difference in diversity between the known and mock samples is seen in V9 for both Family and Genus further confirming that the V9 region does not give reliable results.

## Discussion

A major goal of all human microbiome projects is to identify the bacteria that compose complex communities. In order to do this, valid and reproducible methods are required. The purpose of this paper is to validate and demonstrate the utility of a recently developed method that is capable of identifying bacteria using six hypervariable regions of the 16S rRNA gene. Since the advent of NGS and read length limitations with standard NGS technologies, researchers typically select one or two regions of the 16S rRNA gene. In this paper we present results using six hypervariable regions to attain a comprehensive view of the human microbiome without introducing the inherent biases of selecting only one or two regions of the gene. Other studies have sequenced multiple hypervariable regions on the same samples and have compared the data across the regions in order to investigate if a region performs better than others [14, 19]. Also, one study used third generation sequence or single-molecule real time sequencing to characterize the lung microbiome [45]. To our knowledge, there are no comparable studies using simultaneously processed multiple hypervariable regions of the 16S rRNA gene as described in this paper. The NIH Human Microbiome Project used a mock community to develop their clinical protocols and compared multiple variable regions. In their discussion of the findings, they noted that using multiple regions of the 16S gene would improve results. [26]

We have shown the results of using multiple hypervariable regions of the 16S rRNA gene using four mock communities at both the family and genus level. The four mock communities had genomic material from 20 species of bacteria or 17 genera and families. Fig 4 displays the results of the analysis across the different hypervariable regions. Our results demonstrate that the amplicons targeting the V4 regions and the V2 forward reads identified all 17 family level bacteria while V2 reverse, and V6-7 forward and reverse were almost as consistent. Amplicons targeting the V9 region performed poorly identifying only 3/17 forward and 9/17 reverse. The V8 region for both forward and reverse identified 14/17 of the bacteria. Since V9 performed poorly it should be noted that the total number of reads targeting V9 was at least 14 times lower than the V3 region which had the highest average total read count of all other regions at 76K for forward and reverse. This could suggest that the Ion 16S^™^ Metagenomics Kit does not do well on the end of the 16S rRNA gene. In addition, we provided the results of the sequencing of the mock samples at the family and genus levels. As noted, at the genus level we were not able to identify the genus *Escherichia* but at the family level were able to identify Enterobacteriaceae.

The Ion Torrent PGM produces unpaired bidirectional sequencing reads with a median length of 200–220 bp, therefore the reads were analyzed separately. There is some published literature suggesting that results from both forward and reverse directions are similar and perhaps do not need to be examined separately [28, 46]. Although those studies were performed on different sequencing platforms and also were from paired reads (Illumina HiSeq and MiSeq and the Genome Analyzer IIx) the results could also be the same for the Ion Torrent. We examined this question and found some differences between the forward and reverse reads thus we conclude that we will present both forward and reverse read results in future work.

In summary, the V2, V4 region and V6-7 displays consistent results. We validated these relationships using two different measures. Regions V2, V4 and V6-7 gave the lowest average D_KL_ over all regions and regions and the Shannon Diversity index was the closest to that of the known in V2, V6-7 and V4 respectively. In examining the different mock communities, the Staggered Low results test the lower number operons in the genomic material against the known results. The Staggered Low set had the lowest number of operons with some bacteria at 10^3^ level. *Actinomyces was* detected in hypervariable regions, V4, V6-7 and V8 and *Deinococcus* was detected in all forward hypervariable regions except V3. The same was true of *Enterococcus*, being detected in all regions in the forward reads. These findings demonstrate the ability of our methods to detect bacteria in low abundance. All 17 bacteria were detected in the key three regions except *Actinomyces*. In order to gain more specificity down to the species level, it might be useful to merge consecutive reads from the V2-V3-V4 regions and from the V67-V8 regions since these reads overlap slightly (Fig 3). While doing this would likely prevent the ability to compute bacterial abundance because of double-counting, it could give a snapshot of which species of bacteria are in fact present or absent within a sample. An analysis like this in addition to the analysis presented in this manuscript might give an even more clear idea of the bacterial microbiota in clinical samples for future studies. There are several limitations to this work. To our knowledge, besides an application note reported by the company there are no other published studies using the primers in Ion 16S^™^ Metagenomics Kit [30]. We used genomic DNA from mock samples and not clinical samples which we processed, however, the next step in our research is to use this method with clinical samples. We were unable to combine the forward and reverse reads without compromising the data therefore we analyzed the forward and reverse reads separately. To date we have not found a method to combine OTUs across multiple variable regions, however such a method would greatly increase the efficiency of the analysis especially when dealing with larger numbers of specimens. Some ideas regarding combining data from multiple V regions will be explored in future work, since the ability to generate one result over all regions will greatly increase the efficiency of our analysis especially when we are dealing with larger number of specimens.

## Conclusion

The results of our analysis have shown that our sequencing methods using 6 hypervariable regions of the 16S rRNA and subsequent analysis are valid. We concluded that we still need to examine the forward and reverse reads separately because there are differences (S2 Fig). The V4 hypervariable region performed the best and produced the lowest Kullback-Leibler divergence but V2 and V67 also yielded comparable results. A strategy for combining the results over the multiple variable regions in order to come up with one consensus result will be sought.

## Supporting Information

S1 Fig Incidence Map of Bacterial Presence in Reverse Reads. Incidence map showing whether bacteria was found in the reverse reads at both the family and genus (red), at the family only (blue) or not found at family or genus level (black X) for each of the 6 regions.
(TIF)

S2 Fig Genus Level Bacterial Abundance Chart in Even High and Staggered High Known and Observed Samples. (Fig A) Known Even vs. Even High mock samples at genus level, (Fig B) Known Staggered vs. Staggered High mock samples at genus level.
(TIF)

S3 Fig Kullback-Leibler Divergence for Family and Genus Level in Reverse Reads. Bar graph showing family (upper) and genus (lower) level Kullback-Leibler divergence (y-axis) for 6 regions (x-axis). Known mock compared to observed mock (EvHi, purple; EvLo green; StHi red; StLo blue) for reverse reads. The average D_KL_ over all mock samples for each region is shown above the bar charts for a particular region.
(TIF)

S4 Fig Bivariate Plots of OTU counts for Forward and Reverse for V2, V4 and V6-7. Total OTU counts for forward (y-axis) and reverse (x-axis) reads for 3 regions. (Fig A) Forward and reverse reads for region V2 for four mock samples. (Fig B) Forward and reverse reads for region V4 for four mock samples. (Fig C) Forward and reverse reads for region V6-7 for four mock samples. Each panel shows, even high in the upper left. Even low in the lower left. Staggered high in the upper right. Staggered low in the lower right. Red line denotes the line of identity.
(TIF)
