# Supplementary figures and images for: Development of an Analysis Pipeline Characterizing Multiple Hypervariable Regions of 16S rRNA Using Mock Samples
Source: PLoS One. 2016 Feb 1;11(2):e0148047. doi: 10.1371/journal.pone.0148047 (PMC4734828; doi:10.1371/journal.pone.0148047)

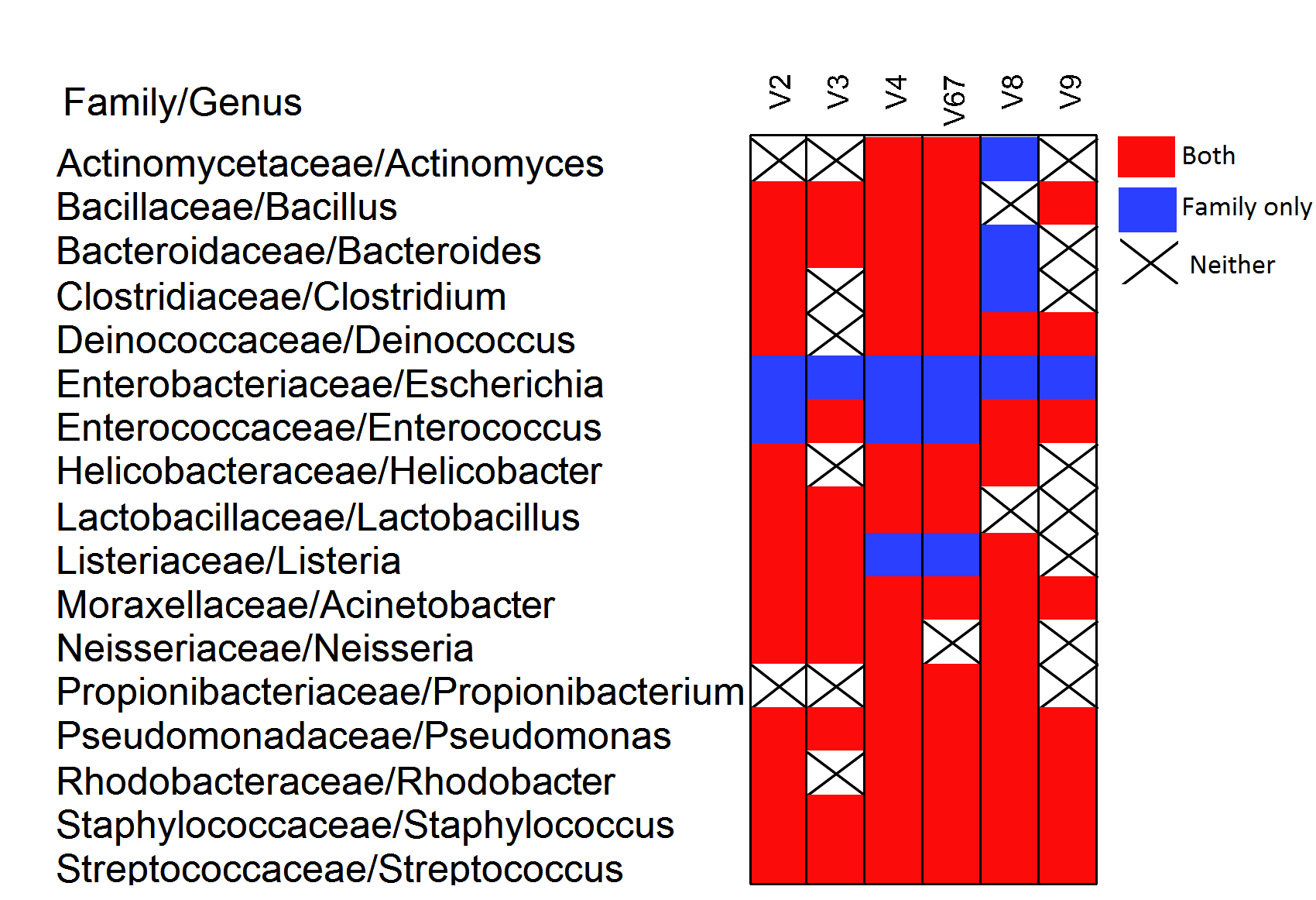

Supplement: S1 Fig — Incidence map showing whether bacteria was found in the reverse reads at both the family and genus (red), at the family only (blue) or not found at family or genus level (black X) for each of the 6 regions. (TIF) [file pone.0148047.s001.tif]

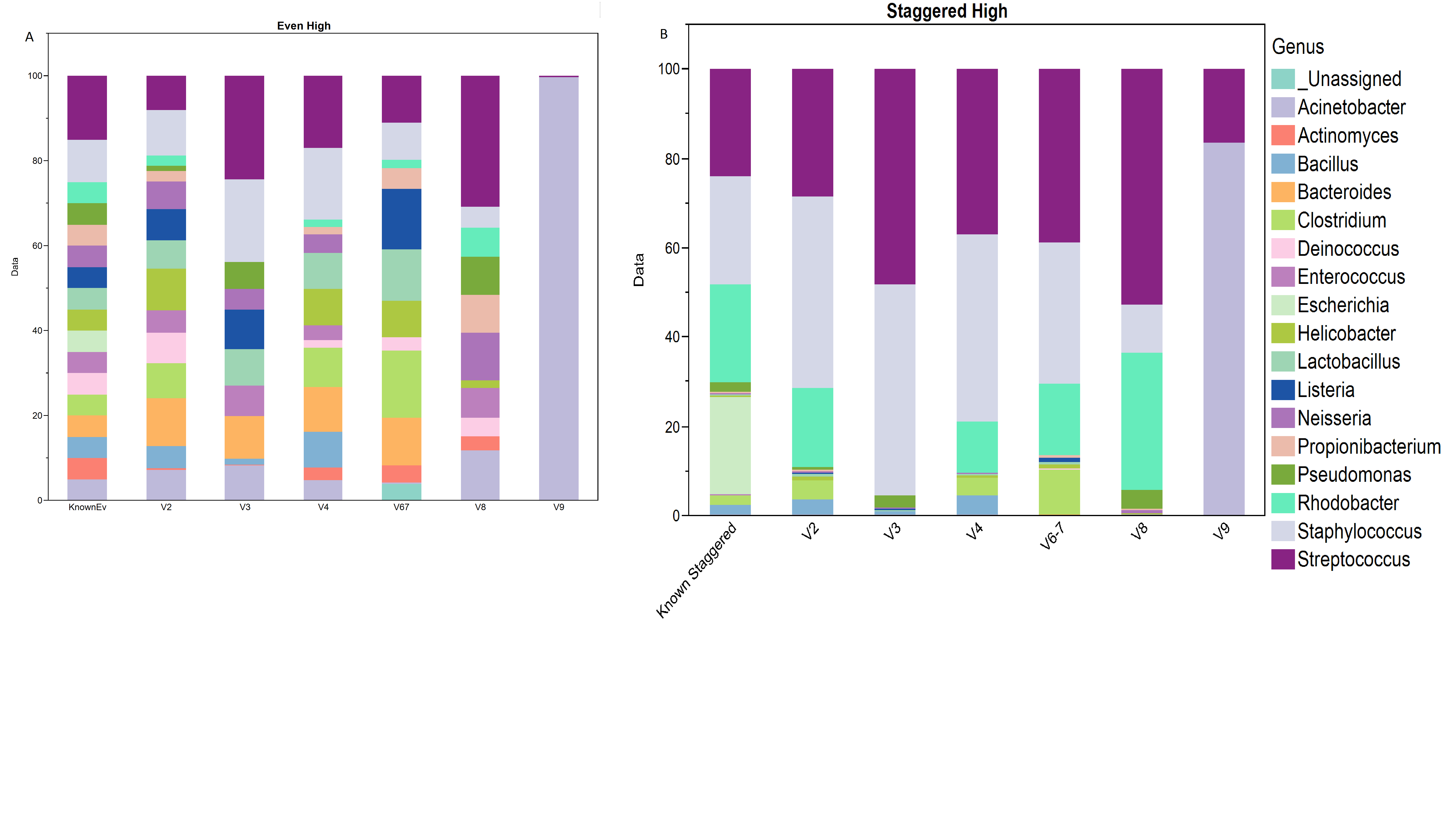

Supplement: S2 Fig — (Fig A) Known Even vs. Even High mock samples at genus level, (Fig B) Known Staggered vs. Staggered High mock samples at genus level. (TIF) [file pone.0148047.s002.tif]

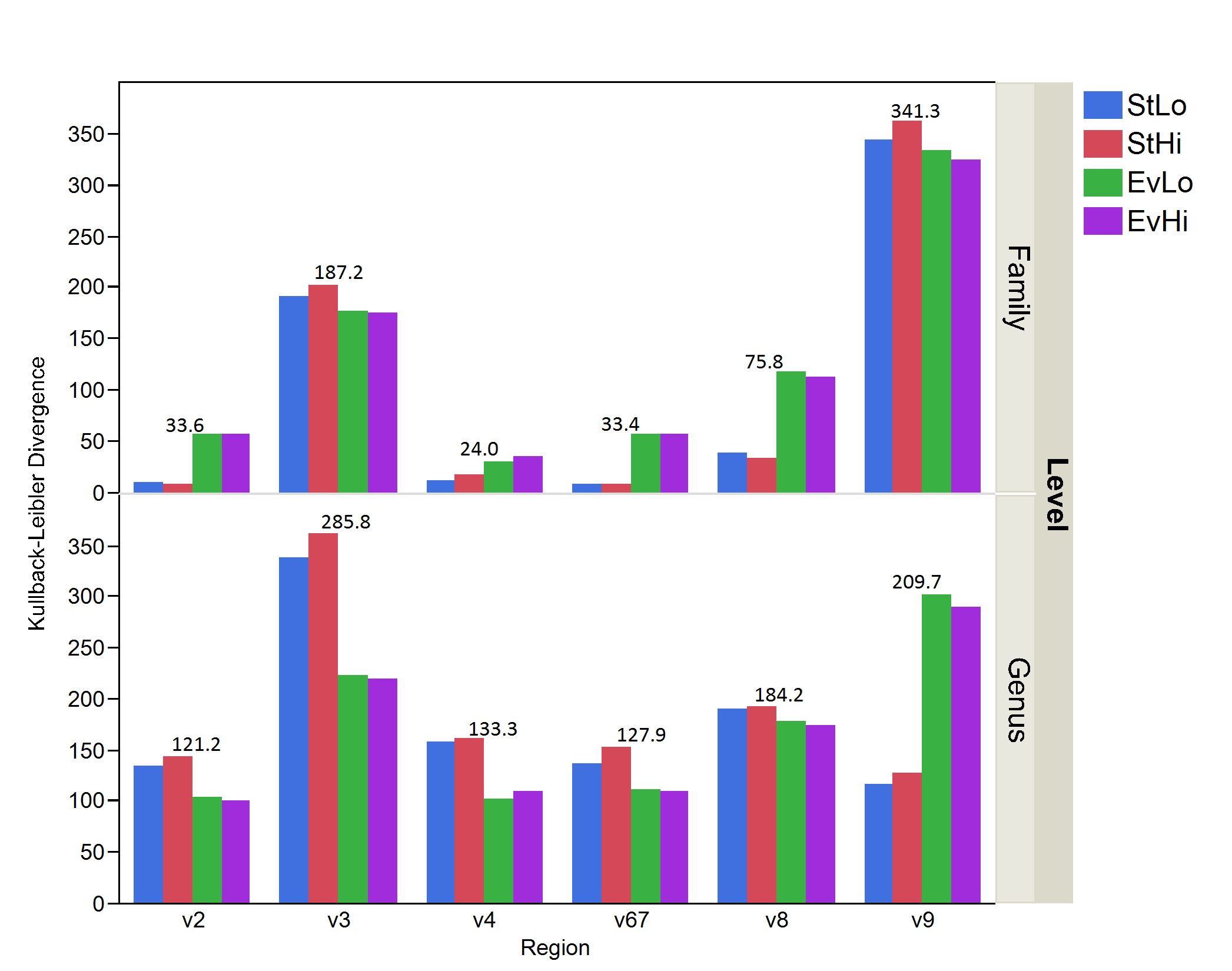

Supplement: S3 Fig — Bar graph showing family (upper) and genus (lower) level Kullback-Leibler divergence (y-axis) for 6 regions (x-axis). Known mock compared to observed mock (EvHi, purple; EvLo green; StHi red; StLo blue) for reverse reads. The average DKL over all mock samples for each region is shown above the bar charts for a particular region. (TIF) [file pone.0148047.s003.tif]

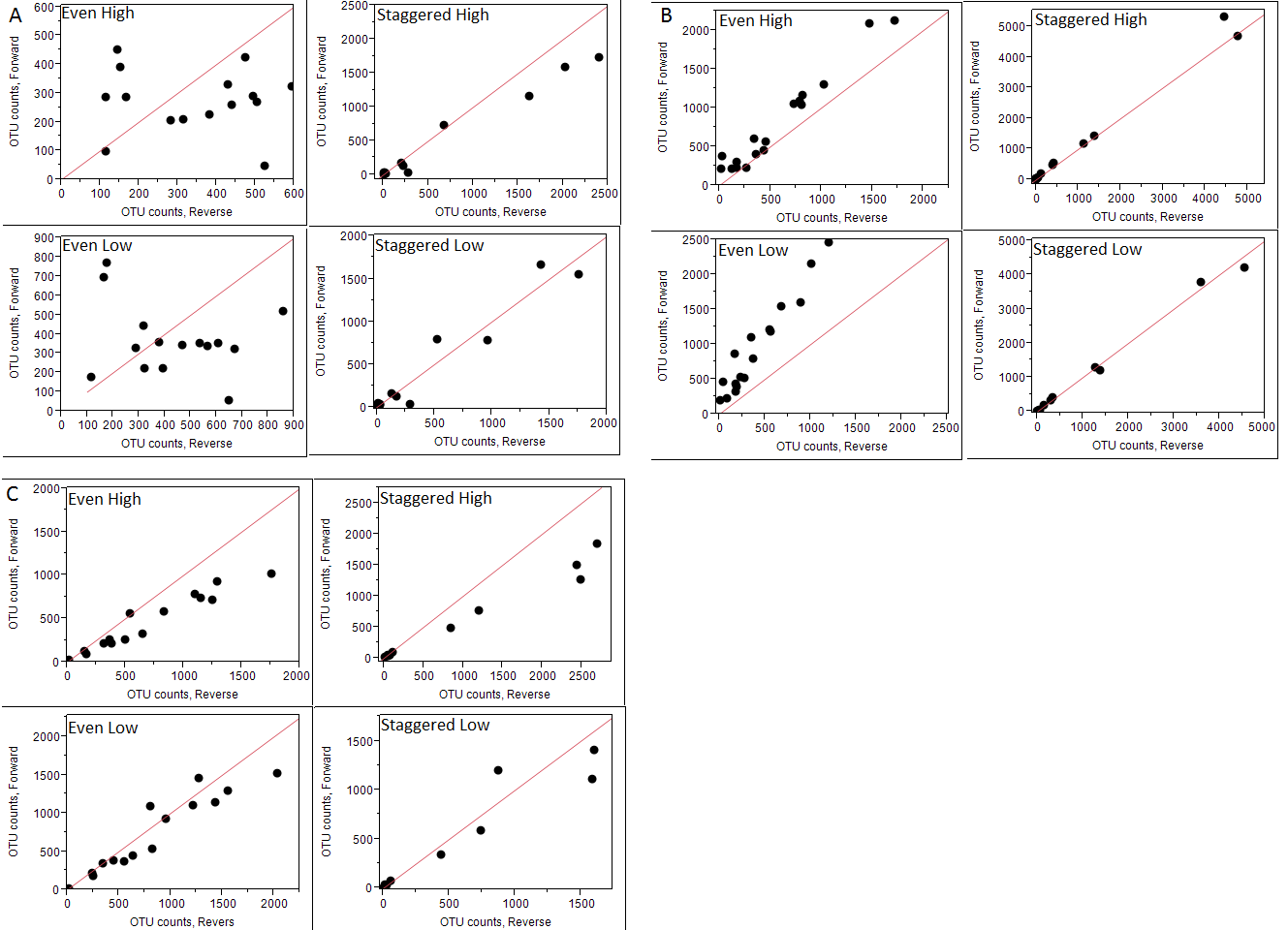

Supplement: S4 Fig — Total OTU counts for forward (y-axis) and reverse (x-axis) reads for 3 regions. (Fig A) Forward and reverse reads for region V2 for four mock samples. (Fig B) Forward and reverse reads for region V4 for four mock samples. (Fig C) Forward and reverse reads for region V6-7 for four mock samples. Each panel shows, even high in the upper left. Even low in the lower left. Staggered high in the upper right. Staggered low in the lower right. Red line denotes the line of identity. (TIF) [file pone.0148047.s004.tif]
